# Impact of awareness of genetic status on cognitive, behavioral, and neuropsychiatric outcomes in genetic frontotemporal dementia

**DOI:** 10.1007/s00415-026-13892-0

**Published:** 2026-06-12

**Authors:** Liset de Boer, Babette Reichard, Jackie M. Poos, Julie F. H. De Houwer, Tine Swartenbroekx, Olaiya A. Aro, Ana Rajicic, Annika Brouwer, Elise G. P. Dopper, Laura Donker Kaat, Harro Seelaar, Lize C. Jiskoot

**Affiliations:** 1 Department of Neurology and Alzheimer Center, Erasmus MC University Medical Center, Dr. Molewaterplein 40, 3015 GD Rotterdam, The Netherlands; 2 Department of Clinical Genetics, Erasmus MC University Medical Center, Dr. Molewaterplein 40, 3015 GD Rotterdam, the Netherlands

**Keywords:** Frontotemporal dementia, Genetics, Cognition, Neuropsychology, Neuropsychiatry

## Abstract

**Background:**

Prior research on genetic frontotemporal dementia (FTD) has examined the psychological impact of predictive testing, leaving other effects of genetic awareness largely unexplored. This study examined whether genetic awareness is associated with cognitive, behavioral, and neuropsychiatric outcomes in individuals from genetic FTD families.

**Methods:**

From a longitudinal genetic cohort study, we included 228 individuals (age 18–70 years) from families with different underlying pathogenic variants. 99 individuals were aware of their genetic status, of which 75 pathogenic variant carriers and 24 non-carriers. 129 individuals were unaware of their genetic status, of which 56 carriers and 73 non-carriers. 26 individuals became aware of their genetic status mid-study. We compared baseline and longitudinal cognitive, behavioral, and neuropsychiatric outcomes between the aware and unaware groups, and explored differences in longitudinal slopes before and after becoming aware of the genetic status.

**Results:**

Cross-sectionally, genetic awareness is associated with the anxiety subdomain of the NPI-Q, but not after FDR correction. Longitudinally, awareness of genetic status was associated with decline in verbal learning, recall, and semantic fluency in carriers. Non-carriers who learned their status mid-study reported fewer depressive symptoms post-disclosure, but depressive symptoms increased over time.

**Conclusion:**

Awareness of genetic status is associated with faster cognitive decline and greater behavioral and neuropsychiatric symptoms in aware carriers of FTD variants. These findings suggest that genetic awareness is an important factor to consider during personalized guidance and monitoring in clinical trials. Future studies should aim for larger sample sizes to further explore potential effects in genetic subgroups.

**Supplementary Information:**

The online version contains supplementary material available at 10.1007/s00415-026-13892-0.

## Introduction

Genetic frontotemporal dementia (FTD) refers to a subset of approximately 10–40% of cases that are caused by autosomal dominant pathogenic variants [1]. The common genetic causes include pathogenic variants in *progranulin* (*GRN*) and *microtubule-associated protein tau* (*MAPT*), as well as a hexanucleotide repeat expansion in *chromosome 9 open reading frame 72* (*C9orf72*) [2]. These variants are highly penetrant but have variable ages of symptom onset, even among individuals within the same family [3]. Multicenter studies such as the Genetic FTD Initiative (GENFI) have been analyzing clinical, cognitive, and biomarker differences between pathogenic variant carriers and non-carriers [2]. The results of this study have led to the identification of early markers of disease onset and progression, providing valuable insights for upcoming clinical trials targeting the preclinical phase.

First-degree family members of genetic FTD patients have a 50% risk of inheriting a pathogenic variant. Some choose to undergo predictive genetic testing [4]. Previous research on this topic has explored decisions to undergo genetic testing and its possible psychological consequences. In the Dutch genetic FTD Risk cohort (FTD-RisC), participants expressed a willingness to receive their genetic test results, particularly when this was linked to opportunities for clinical trial participation [5, 6]. Many also noted that learning their genetic status would help them prepare for the future by arranging appropriate care or considering early retirement. At the same time, individuals acknowledged that disclosure of a positive genetic result might have adverse psychological effects, potentially affecting self-perception and interpersonal relationships [5, 6].

The psychological effects of learning one’s genetic status have been studied in FTD and other neurodegenerative diseases [7–11]. In genetic FTD, predictive testing appears to cause no long-term psychological harm and does not negatively affect social or personal functioning [7–9]. Similarly, studies on Alzheimer’s disease (AD) have reported minimal psychological risk following disclosure of the homozygous apolipoprotein E (*APOE*) ε4 genotype, a variant associated with increased risk for developing AD [10–12]. However, learning one’s genetic status was found to lead to increased test-related distress and a short-term increase in depressive symptoms [12]. Moreover, a study on the psychological effects of genetic testing in Huntington’s disease (HD) showed short-term increases in psychological distress in carriers [13]. Negative genetic testing results provided relief and did not lead to life changes [5–7]. Together, these findings suggest that although genetic risk disclosure is not usually associated with long-term psychological difficulties, it can elicit short-term emotional responses that may be relevant for clinical monitoring and research.

The effect of awareness of genetic status on cognitive performance has been studied previously in individuals with autosomal dominant AD [12]. The study findings indicated that awareness of being a carrier did not influence the rate of cognitive decline but was associated with lower cognitive composite scores at the post-disclosure visit in comparison to carriers who remained unaware of their genetic status. Among aware non-carriers, awareness of status had no relationship with depressive symptoms, clinical status, or cognition. Similarly, Lineweaver et al. [14] found that carriers who were aware of their genetic status rated their memory more negatively and performed worse on verbal memory tests than those who were unaware of their status. How such effects manifest in genetic FTD has not been investigated yet. If knowledge of one’s genetic status influences behavior or cognitive performance, it may complicate the interpretation of outcomes in research studies, clinical trials, and routine clinical practice, where such measures are used to guide counseling, monitoring, and diagnostic decision-making. Aware carriers may report more symptoms, experience greater anxiety during neuropsychological testing, or perform differently on cognitive tests due to expectancy [12], while unaware carriers may not. This could cause systematic differences unrelated to underlying pathology.

To address these concerns, we examined cognitive, behavioral, and neuropsychiatric outcomes in individuals from genetic FTD families who were symptom free and either aware or unaware of their genetic status. We analyzed baseline differences as well as longitudinal effects. Additionally, we analyzed outcomes among participants who discovered their status during the study period to assess the impact of learning one’s genetic status after study enrollment.

## Methods

### Participants

**Fig. 1:**
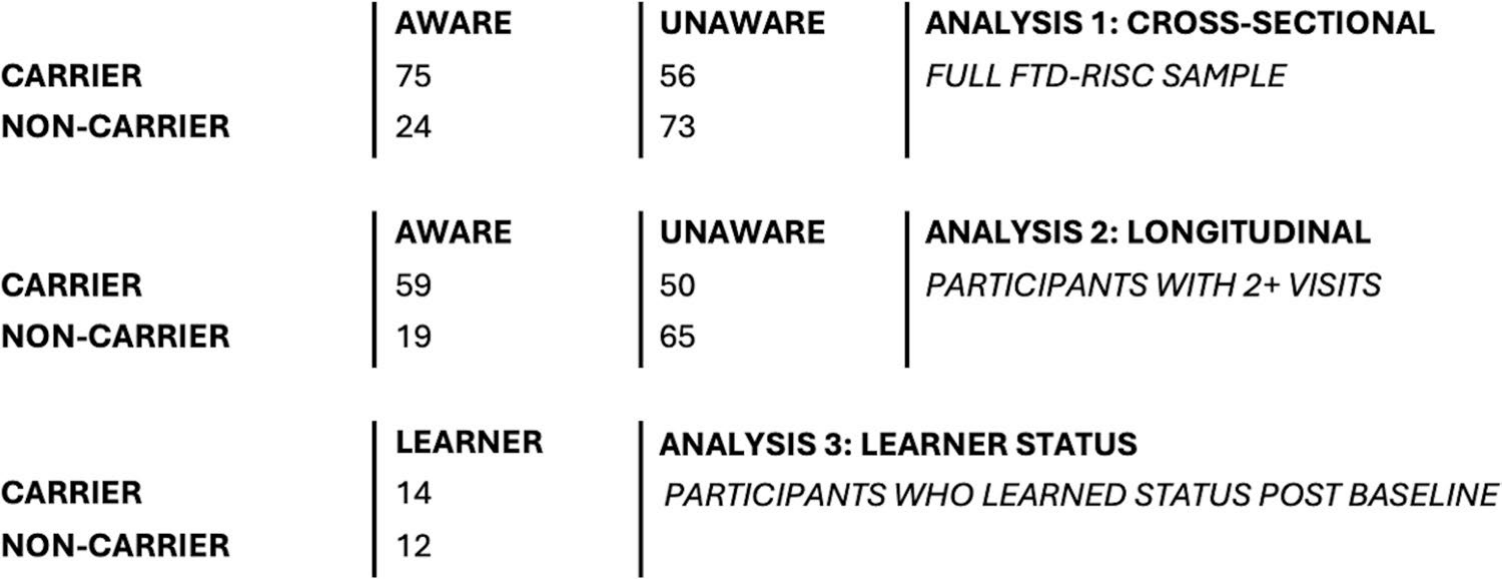
Overview of participants in the three analyses. Unaware = unaware of genetic status, aware = aware of genetic status, learner = became aware of genetic status mid-study

The study included all eligible participants (*n* = 228) of the FTD-RisC study from the Erasmus University Medical Center (Rotterdam, The Netherlands), a study in which first-degree family members of patients with genetic FTD are followed longitudinally [15]. Data were gathered between May 2010 and November 2025. Genetic testing distinguished carriers with different pathogenic variants (total *n* = 131; *C9orf72* *n* = 58, *GRN* *n* = 49, *MAPT* *n* = 19, *TARDBP* *n* = 4, *TUBA4A* *n* = 1) from non-carriers (*n* = 98). Longitudinal data (2–13 study visits) were available for a total of 193 participants (Fig. 1). Participants were *aware* or *unaware* of their genetic status. Aware participants underwent genetic testing at their own request and were divided into the following four groups: (1) *aware carriers* (aware at study baseline), (2) *aware non-carriers*, (3) *learner carriers* (becoming aware after study baseline), or (4) *learner non-carriers*. Unaware participants were divided into (1) *unaware carriers* or (2) *unaware non-carriers* (Fig. 1). All participants were presymptomatic and had a global Clinical Dementia Rating scale plus National Alzheimer’s Coordinating Center Frontotemporal Lobar Degeneration Behavior and Language domains (CDR^®^-plus-NACC-FTLD) [16] score of 0 (normal) or 0.5 (mild impairment). Prodromal participants (defined as those with a global CDR^®^-plus-NACC-FTLD score of 0.5 across multiple study visits without reverting to 0) and fully symptomatic participants at baseline (CDR^®^-plus-NACC-FTLD ≥ 1) were excluded to avoid confounding by overt disease effects. For carriers who progressed during the study, only visits at which they remained asymptomatic (CDR^®^-plus-NACC-FTLD = 0) were included. Visits with a CDR^®^-plus-NACC-FTLD score of 0.5 were included only if this occurred at a single visit and reverted to 0 at subsequent follow-up, indicating no sustained prodromal progression. Visits showing persistent or increasing scores of 0.5 or higher were excluded.

### Procedure

All participants underwent a standardized clinical assessment, consisting of a structured interview with the participant and a knowledgeable informant (including the CDR^®^-plus-NACC-FTLD [16]), a neurologic examination, a neuropsychological assessment, and a T1-weighted 3T brain MRI.

#### Neuropsychological assessment

Global cognitive functioning was screened using the Mini-Mental State Examination (MMSE) [17] and Montreal Cognitive Assessment (MoCA) [18]. As some participants completed both the MoCA and the MMSE, while others only completed the MMSE or the MoCA, MoCA scores were converted to MMSE-equivalent scores to ensure consistency across participants (based on the weighted equivalence formula by Fasnacht et al. [19]). For memory, we conducted the Rey Auditory Verbal Learning Test (AVLT) Dutch version [20]. We used the Boston Naming Test (BNT—60 items) [21] and the semantic and letter fluency tests to assess language. For assessment of attention and mental processing speed, we used Trail Making Test (TMT) part A and Stroop Color Word Test (SCWT) card I and II. For executive functioning, we used TMT part B [22] and SCWT card III [23]. Social cognition was assessed using the Emotion Recognition Test (ERT) [24] and the facial emotion recognition part of the mini-SEA [25]. For visuoconstruction, we used the Clock Drawing Test [26]. Parallel versions of the letter fluency task (3 versions) and the AVLT (4 versions) were used to reduce familiarity and practice effects as much as possible.

#### Behavioral and neuropsychiatric assessment

Beck’s Depression Inventory-II (BDI-II) was included to assess the presence and severity of depressive symptoms [27]. This self-report questionnaire contains 21 multiple-choice questions about cognitive, affective, and somatic symptoms. Each item is scored on a scale from 0 to 3, with higher scores indicating more severe symptoms [27]. We analyzed behavioral changes and functional decline using the Frontotemporal Dementia Rating Scale [28]. This caregiver questionnaire evaluates seven aspects of a patient’s personality and daily activities on a 3-point scale reflecting the frequency of specific impairments. Scores were converted into percentages, with a lower percentage indicating greater impairment of everyday abilities [28]. The Neuropsychiatric Inventory Questionnaire (NPI-Q) [29] was used to assess the behavioral and psychological symptoms commonly seen in neurological disorders [29]. This caregiver questionnaire contains 12 domains. Each symptom is rated for frequency (score ranging from 1 to 4) and severity (score ranging from 1 to 3). A higher score reflects more symptoms. Additionally, we used the Cambridge Behavioral Inventory-Revised (CBI-R), an 81-item, informant-based questionnaire, to measure cognitive, behavioral, affective symptoms, and activities of daily living across 13 domains [30]. Each behavior is rated on a 0–4 scale, with higher scores reflecting more severe deficits. The total scores of the BDI-II, NPI-Q, and CBI-R, the percentage of the FRS, and the scores on the 12 subdomains of the NPI-Q (delusions, hallucinations, agitation/aggression, depression/dysphoria, anxiety, elation/euphoria, apathy/indifference, disinhibition, irritability/lability, aberrant motor behavior, nighttime behavior, appetite/eating) were included in the statistical analysis.

### Statistical analysis

Statistical analyses were performed using R (v4.3.1, R Foundation for Statistical Computing, Vienna, Austria) with tidyverse tools and the packages *lme4* and *rmcorr*. The significance level was set at *p* < 0.05 (2-tailed) across all comparisons, and we implemented Benjamini–Hochberg false discovery rate (FDR) corrections for multiple testing. We compared continuous demographic data between groups with linear models. We analyzed categorical variables with Chi-square tests. We compared clinical and cognitive data between asymptomatic carriers and non-carriers (both aware and unaware) with classical linear models, corrected for sex, age, and education. Baseline differences were visualized with the package *ggplot2*. Longitudinal trends were visualized with the package *ggplot2* using the LOESS method.

#### Analysis 1: cross-sectional analysis

To examine the effect of awareness of the genetic status at baseline, we divided the participants into carriers and non-carriers and performed a linear model with genetic awareness, age, sex, and education as covariates. Among individuals aware of their genetic status, years since genetic disclosure were additionally included as a covariate. In this analysis and analysis 2 (below), we included the first post-disclosure visit for the *learner* group, so that they could be added to the *aware* group. To assess the robustness of the findings, a sensitivity analysis excluding the *learner* group was also performed. To explore potential differences between pathogenic variants, we ran another model with an interaction effect of genetic subgroup and awareness.

#### Analysis 2: longitudinal analysis

To examine the effect of awareness of genetic status over time, we created separate linear mixed models for carriers and non-carriers with all fixed effects (baseline age, sex, education, time, genetic awareness, years since genetic disclosure), a two-way interaction between time (follow-up data) and genetic awareness (aware vs. unaware), and subject as random effect for both carriers and non-carriers. Participants with only one timepoint were removed from this analysis. For the AVLT and the letter fluency task, we performed an additional analysis including parallel version as a covariate. To address potential bias due to sparse data at later follow-up visits, and due to the significant difference in number of follow-up visits between status, we performed a sensitivity analysis restricting the dataset to participants with a maximum of seven follow-up visits. Under this restriction, the differences between status groups were no longer statistically significant. A sensitivity analysis excluding the *learner* group was also performed.

To examine whether greater neuropsychiatric symptoms were associated with cognitive performance, we conducted a repeated-measures correlation analysis between BDI-II scores and the cognitive outcomes on which awareness had a significant effect. We used the BDI-II for this analysis as it is the only self-reported measure of neuropsychiatric symptoms in the dataset and showed the greatest variation across groups compared with the NPI-Q and the FRS.

#### Analysis 3: learner status

First, we performed a paired *t*-test to examine the differences between outcomes of the last visit before and the first visit after learning the genetic status in individuals who learned it mid-study. Secondly, using the longitudinal data, we fitted a model in which time was partitioned into two segments: before and after learning the genetic status. As the random component did not improve model fit and led to instability, we proceeded with a fixed-effects linear model. Time (in years from baseline), learning status (0 = before learning, 1 = after learning), and their interaction with status (carrier, non-carrier) were included, along with covariates for age, sex, education, and parallel version—where applicable. This method allows the slope of change to differ before versus after learning genetic status, enabling direct comparison of trajectories across segments. To address potential bias due to sparse data at later follow-up visits, we performed sensitivity analyses restricting the dataset to a maximum of three visits before learning and three visits after learning the genetic status.

## Results

### Demographics

Baseline demographics, cognitive test scores, and questionnaire scores are presented in Table 1. There were no significant differences in demographic data between carriers and non-carriers (both aware and unaware). Carriers had a significantly higher CDR^®^-plus-NACC-FTLD sum of boxes score [*β* = 0.25, 95% CI 0.02–0.48, SE = 0.12, *p* = 0.03]. There was a significant difference in gene distribution between aware and unaware carriers [*χ*^2^(4) = 17.44, *p* = 0.002]. In addition, compared to non-carriers, carriers had significantly lower baseline performances on category fluency (occupations) [*β* = − 2.25, 95% CI − 3.48 to − 1.03, SE = 0.62, *p*_corrected_ = 0.003] and Stroop card II [*β* = 4.61, 95% CI 1.43–7.77, SE = 1.61, *p*_corrected_ = 0.0]. There were no significant differences in the questionnaires.

**Table 1:** Baseline demographics

	Carriers			Non-carriers		
	Aware	Unaware	*p*-value	Aware	Unaware	*p*-value
*N*	75	56	NA	24	73	NA
Sex (M/F)	29/46	25/31	0.61	9/15	34/39	0.59
Age (years)	46.30 ± 12.50	44.8 ± 12.40	0.50	47.70 ± 12.80	47.30 ± 12.10	0.88
Education (years)	14.10 ± 2.75	14.20 ± 2.55	0.09	14.90 ± 2.49	13.90 ± 3.19	0.13
FTD CDR sum of boxes	0.36 ± 0.84	0.08 ± 0.34	0.24	0.00 ± 0.00	0.05 ± 0.28	0.64
Genotype			**< 0.01**	NA	NA	NA
*C9*	42	16				
*GRN*	17	32				
*MAPT*	13	6				
*Other*	3	2				
Time since genetic disclosure	1.75 (3.34)	NA	NA	1.48 (2.19)	NA	NA
Median number of follow-ups (min–max)	4 (1–12)	6 (1–12)	**0.03**	4 (1–10)	6 (1–12)	NA
MMSE conversion score (0–30)	29.13 ± 1.09	28.73 ± 1.73	0.28	28.81 ± 1.30	28.99 ± 1.27	0.43
BNT (0–60)	53.67 ± 4.71	53.23 ± 4.39	0.59	56.25 ± 3.28	53.57 ± 4.20	0.31
Animal fluency (number of words)	24.97 ± 5.44	23.85 ± 5.55	0.20	27.46 ± 5.58	25.33 ± 6.44	0.68
Occupations fluency (number of words)	17.45 ± 4.39	17.24 ± 5.07	0.74	20.25 ± 4.37	19.28 ± 5.17	0.76
Letter fluency (number of words)	36.04 ± 10.88	36.43 ± 11.54	0.96	44.21 ± 13.13	36.39 ± 12.31	0.31
Stroop I (s)	47.64 ± 9.31	45.42 ± 9.18	0.35	43.67 ± 5.63	45.64 ± 8.24	0.36
Stroop II (s)	60.83 ± 14.98	59.60 ± 13.19	0.95	55.29 ± 9.46	56.44 ± 10.69	0.89
Stroop III (s)	89.39 ± 23.87	92.60 ± 23.59	0.42	82.67 ± 18.00	88.97 ± 35.30	0.58
TMT A (s)	30.11 ± 10.95	29.38 ± 11.03	0.81	25.80 ± 8.71	30.18 ± 12.72	0.39
TMT B (s)	66.07 ± 27.60	67.41 ± 35.79	0.76	58.25 ± 13.74	69.49 ± 34.91	0.43
AVLT learning (0–75)	46.93 ± 9.80	47.35 ± 11.28	0.96	49.63 ± 12.05	46.24 ± 10.39	0.43
AVLT delayed recall (0–15)	9.90 ± 2.97	9.50 ± 3.30	0.57	10.38 ± 3.62	9.30 ± 3.43	0.61
AVLT recognition (0–30)	28.92 ± 1.77	29.15 ± 1.19	0.96	28.25 ± 3.29	29.00 ± 1.78	0.37
Clock drawing (0–14)	12.26 ± 1.25	12.36 ± 1.30	0.77	12.54 ± 1.06	12.49 ± 1.43	0.96
Mini-SEA FER total score (0–35)	28.69 ± 2.46	31.00 ± 0.82	0.25	29.25 ± 2.06	29.00 ± 3.35	0.50
ERT total score (0–90)	58.74 ± 7.77	60.08 ± 8.12	0.78	62.15 ± 7.28	61.69 ± 7.54	0.96
BDI (0–63)	5.24 ± 7.00	3.43 ± 4.29	0.58	2.95 ± 3.28	4.07 ± 4.60	0.70
FRS (0–100)	91.69 ± 16.54	95.15 ± 9.94	0.82	97.92 ± 3.54	95.59 ± 17.72	0.93
NPI-Q (0–144)	4.22 ± 12.35	1.25 ± 2.84	0.35	0.38 ± 1.12	0.92 ± 2.93	0.88
*Agitation (0–12)*	0.43 ± 1.70	0.30 ± 1.32	0.88	0.00 ± 0.00	0.07 ± 0.34	0.83
*Anxiety (0–12)*	0.27 ± 1.11	0.00 ± 0.00	0.15	0.00 ± 0.00	0.02 ± 0.15	0.98
*Apathy (0–12)*	0.75 ± 2.65	0.15 ± 0.48	0.80	0.00 ± 0.00	0.07 ± 0.26	0.77
*Appetite (0–12)*	0.55 ± 2.53	0.00 ± 0.00	0.18	0.00 ± 0.00	0.21 ± 0.87	0.93
*Delusions (0–12)*	0.16 ± 0.91	0.00 ± 0.00	0.43	0.00 ± 0.00	0.00 ± 0.00	NA
*Depression (0–12)*	0.88 ± 2.69	0.15 ± 0.48	0.19	0.08 ± 0.28	0.24 ± 0.88	0.91
*Disinhibition (0–12)*	0.16 ± 0.68	0.00 ± 0.00	0.96	0.00 ± 0.00	0.00 ± 0.00	NA
*Elation (0–12)*	0.18 ± 0.84	0.13 ± 0.52	0.62	0.00 ± 0.00	0.02 ± 0.15	0.98
*Hallucinations (0–12)*	0.00 ± 0.00	0.03 ± 1.16	0.58	0.00 ± 0.00	0.00 ± 0.00	NA
*Irritability (0–12)*	0.50 ± 1.91	0.38 ± 1.08	0.94	0.15 ± 0.38	0.17 ± 0.66	0.70
*Motor behavior (0–12)*	0.00 ± 0.00	0.00 ± 0.00	NA	0.00 ± 0.00	0.05 ± 0.38	0.94
*Night behavior (0–12)*	0.39 ± 1.90	0.13 ± 0.65	0.51	0.15 ± 0.55	0.02 ± 0.15	0.14
CBI-R (0–180)	5.85 ± 8.57	3.36 ± 6.75	0.71	2.93 ± 5.03	2.93 ± 3.76	0.99

Values indicate mean ± SD

*CDR* Clinical Dementia Rating scale, *C9orf72* chromosome 9 open reading frame 72, *GRN* progranulin, *MAPT* microtubule-associated protein tau, *MMSE* Mini-Mental State Examination, *BNT* Boston Naming Test, *TMT* Trail Making Test, *AVLT* Auditory Verbal Learning Test, *FER* Facial Emotion Recognition, *ERT* Emotion Recognition Test, *BDI* Beck’s Depression Inventory, *FRS* Frontotemporal Dementia Rating Scale, *NPI-Q* Neuropsychiatric Inventory Questionnaire, *CBI-R* Cambridge Behavioral Inventory-Revised

*p*-values of cognitive, behavioral, and neuropsychiatric measures are adjusted for age, sex, education, years since genetic disclosure, and multiple testing (FDR). *p*-values < 0.05 are displayed in bold

#### Analysis 1: cross-sectional analysis

**Fig. 2:**
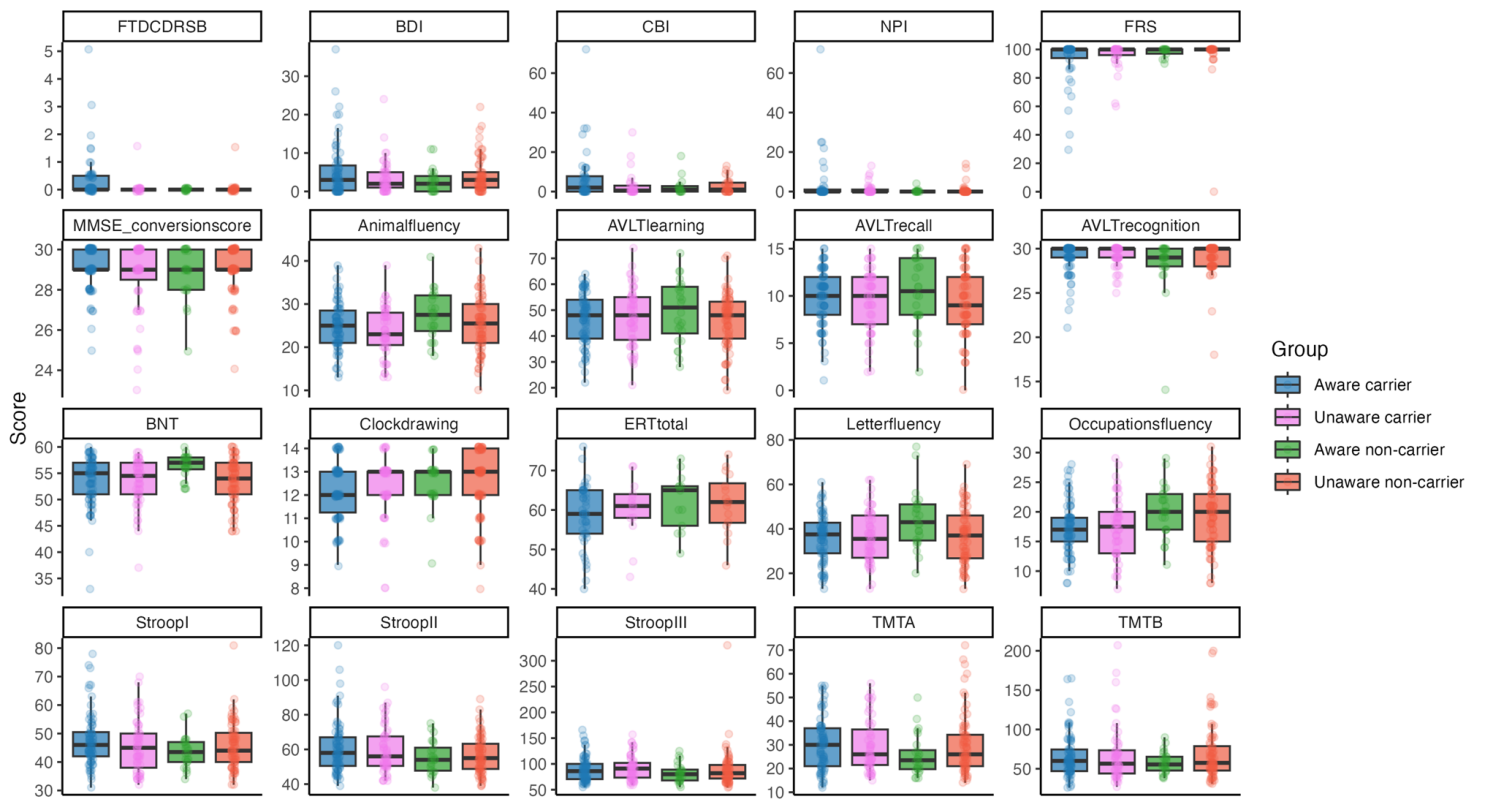
Baseline scores (mean, standard deviation) on cognitive, behavioral, and neuropsychiatric outcomes in all groups

In carriers*,* genetic awareness significantly associated with the anxiety subdomain of the NPI-Q [*β* = 0.37, 95% CI 0.01–0.74, SE = 0.18, *p* = 0.04] (Suppl. Figure 1), but this effect did not remain significant after FDR correction [*p*_corrected_ > 0.1] and after excluding participants from the *learner* group in the sensitivity analysis (*n* = 117)*.* There was no interaction effect of status and genetic subgroup on cognitive, behavioral, or neuropsychiatric outcomes. In non-carriers*,* genetic awareness was associated with higher scores on the nighttime behavior subdomain of the NPI-Q [*β* = 0.28, 95% CI 0.03–0.55, SE = 0.13, *p* = 0.03], but this effect did not remain significant after FDR correction [*p*_corrected_ > 0.1] and after excluding the *learner* group (Fig. 2).

#### Analysis 2: longitudinal analysis

**Fig. 3:**
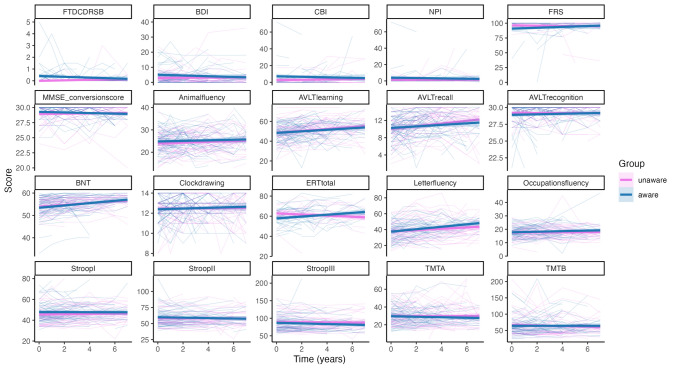
Longitudinal trajectories (95% CI) on cognitive, behavioral, and neuropsychiatric outcomes in carriers

There was a significant interaction between time and genetic awareness on the animal fluency [*β* = − 0.22, 95% CI − 0.42 to 0.01, SE = 0.10, *p* = 0.04], AVLT learning [*β* = − 0.53, 95% CI − 0.9 to 0.14, SE = 0.20,* p* < 0.01], and AVLT recall [*β* = − 0.15, 95% CI − 0.27 to 0.04, SE = 0.06, *p* < 0.01], indicating that aware carriers showed a steeper slope of decline than unaware carriers. The AVLT learning and recall remained significant after FDR correction (*p*_corrrected_ = 0.03 and 0.04, respectively), but not after removing the *learner* group from the analysis and after controlling for parallel versions (all *p*_corrected_ > 0.05). Among these measures, AVLT recall showed a significant correlation with BDI-II scores, such that lower AVLT recall performance was associated with higher levels of depressive symptoms [*r* = − 0.17, 95% CI − 0.31 to 0.03, *p* = 0.02,]*.* In addition, there was a significant interaction between time and genetic awareness on the aberrant motor behavior subdomain of the NPI-Q [*β* = 0.10, 95% CI 0.04–0.16, SE = 0.03, *p*_corrected_ < 0.01] (Suppl. Figure 2), which remained significant after removal of the *learner* group (*p*_corrected_ = 0.02) and was also significantly associated with the BDI-II [*r* = 0.37, 95% CI 0.18–0.54, *p* < 0.01]. Moreover, after removal of the *learner* group, we found a significant interaction between time and genetic awareness on the NPI irritability subdomain [*β* = 0.09, 95% CI 0.03–0.15, SE = 0.03, *p*_corrected_ = 0.03]. In the sensitivity analysis, restricting the dataset to participants with a maximum of seven follow-up visits, there was a significant interaction between time and genetic awareness on the AVLT learning [*β* = − 0.64, 95% CI − 1.12 to 0.04, SE = 0.30, *p* = 0.03], AVLT recall [*β* = − 0.22, 95% CI − 0.40 to 0.03, SE = 0.09, *p* = 0.02], and the ERT total score [*β* = 0.89, 95% CI 0.15–1.66, SE = 0.39 *p* = 0.02] (Fig. 3), which did not remain significant after FDR correction.

**Fig. 4:**
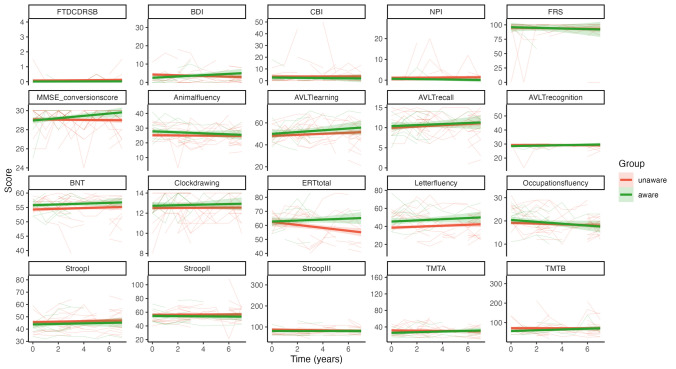
Longitudinal trajectories (95% CI) on cognitive, behavioral, and neuropsychiatric outcomes in non-carriers

In non-carriers, there was a significant interaction between time and genetic awareness and depressive symptoms measured with the BDI-II [*β* = 0.46; 95% CI 0.15–0.77, SE = 0.16, *p*_corrected_ = 0.02], with higher scores in unaware non-carriers at baseline, but higher increases in scores in aware non-carriers over time. In the sensitivity analysis, restricting the dataset to participants with a maximum of seven follow-up visits, the interaction effect of the BDI-II remained significant [*β* = 0.58; 95% CI 0.19–0.97, SE = 0.20, *p*_corrected_ = 0.03] (Fig. 4). This effect did not remain significant after removing the *learner* group from the analysis *(p* > 0.05)*.* After removal of the *learner* group, we also found a significant interaction between time and genetic awareness on the AVLT learning [*β* = 1.16; 95% CI 0.44–0.89, SE = 0.37, *p*_corrected_ = 0.01], indicating higher AVLT learning scores over time. This effect remained significant after controlling for parallel version [*β* = 0.95, 95% CI 0.23–1.67, SE = 0.36, *p*_corrected_ = 0.02]. There were no significant effects on the NPI-Q subdomains (Suppl. Figure 3).

#### Analysis 3: learner status

**Fig. 5:**
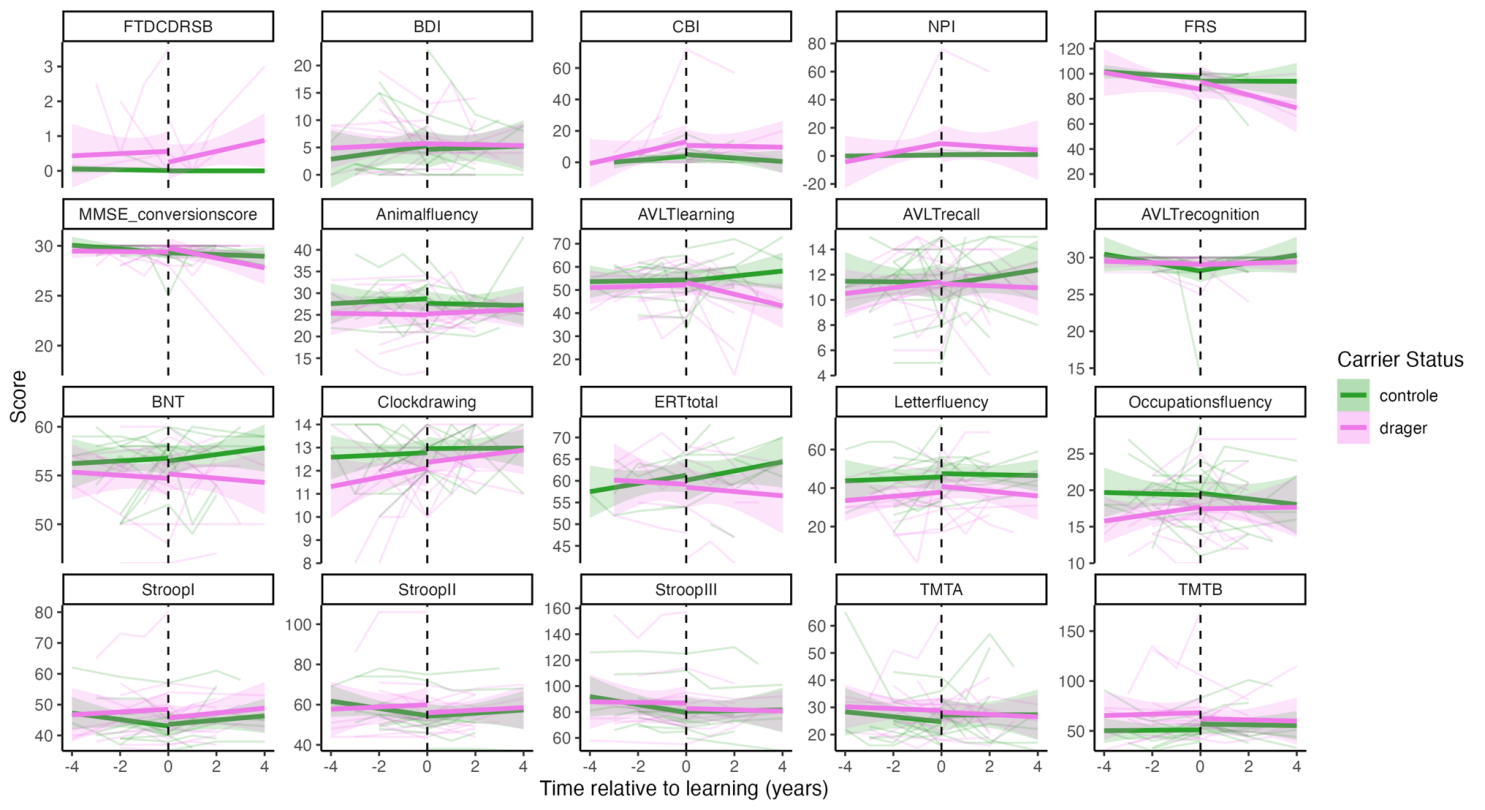
Trajectories (95% CI) pre- and post-awareness on cognitive, behavioral, and neuropsychiatric outcomes in learners with a maximum of 3 follow-ups pre- and -post learning genetic status

In carriers, there were no significant differences in outcomes pre-and post-learning of genetic status. In non-carriers, the paired *t*-test showed a significant difference in BDI-II scores [*t*(8) = 2.47, 95% CI 0.32–9.24, *p* = 0.04], with higher scores before awareness of genetic status compared to after. There was a significant interaction effect of time, learned status, and awareness on the hallucination subdomain of the NPI-Q [*β* = − 0.07; 95% CI 0.11–0.03, SE = 0.02, *p*_corrected_ < 0.01] (Suppl. Figure 4) and the Stroop III [*β* = − 4.86; 95% CI − 7.99 to 1.74, SE = 1.58, *p*_corrected_ = 0.02], indicating that change over time in these scores after learning genetic status differs between carriers and non-carriers. In the sensitivity analysis, restricting the dataset to participants with a maximum of three follow-up visits before and three follow-up visits after learning, these effects did not remain significant (*p*_corrected_ > 0.05) (Fig. 5).

## Discussion

This study examined the impact of genetic awareness on cognitive, behavioral, and neuropsychiatric outcomes in genetic FTD. Cross-sectionally, awareness was not associated with outcomes, but longitudinally it was associated with change in several domains. Among aware carriers, awareness was associated with a steeper decline in verbal learning, verbal recall, and semantic fluency, whereas aware non-carriers showed increasing depressive symptoms over time. In those who learned their status mid-study, non-carriers reported fewer depressive symptoms immediately after disclosure, but no sustained differences in trajectories before versus after awareness were observed. Together, these findings suggest that the impact of awareness of genetic status is subtle, domain-specific, and differs between carriers and non-carriers.

Our longitudinal data revealed effects of genetic awareness on cognitive trajectories over time. Carriers who were aware of their genetic status showed a steeper decline in verbal learning, recall, and semantic fluency compared to unaware carriers. The findings are in line with previous studies on AD [12, 14], but direct comparisons between genetic AD and FTD are warranted as their cognitive profiles and disease progression differ. There is evidence that both verbal memory tests and fluency tasks can be influenced by stress and emotional pressure, which may distort their measurement of pure cognitive ability [31, 32]. We could partially confirm this by finding a weak but significant association between lower performance on the AVLT and increased scores on the BDI-II. These results have significant consequences for cognitive assessment and monitoring in clinical trials, as they highlight that awareness of genetic status may influence both cognitive trajectories. This underscores the importance of providing psychological support and personalized counseling around genetic disclosure. Moreover, at this moment, results from cognitive and clinical assessments should always be interpreted together with other objective measures, such as brain MRI and fluid biomarkers, especially when including presymptomatic individuals in clinical trials, to ensure that subtle subjective effects do not bias trial inclusions and study outcomes [33].

Another explanation for our findings might be that the CDR^®^-plus-NACC-FTLD sum of boxes is higher in aware carriers than unaware carriers, reflecting more clinical symptoms. Importantly, this difference may not necessarily reflect true cognitive decline but could be influenced by *rater bias* during the assessment process. Since clinicians know the participant’s awareness of their genetic status, they may unintentionally rate symptoms as more severe or interpret ambiguous behaviors as signs of decline [34]. Similarly, aware carriers and informants who know about the genetic risk may over-report symptoms. Furthermore, carriers may be more likely to undergo genetic testing after noticing cognitive changes in themselves, meaning they could already be closer to disease onset at study entry [12]. This is supported by the observation that more carriers than non-carriers have undergone genetic testing, although this difference may also be attributable to lower motivation to participate in research among non-carriers. Moreover, the aware group consisted of significantly more *C9orf72* carriers, whereas the unaware group had more *GRN* carriers, and disease onset is particularly difficult to estimate in *C9orf72* carriers [35]. Although all participants remained presymptomatic over time and visits from individuals who developed symptoms during follow-up were excluded from analyses, we cannot fully exclude the possibility that subtle, preclinical changes influenced observed trajectories [36]. Sensitivity analyses restricting follow-up duration yielded similar patterns, but these approaches cannot entirely disentangle early disease-related changes from the psychological impact of awareness. These findings underscore the need for future studies to disentangle true cognitive decline from pre-existing clinical differences and awareness-related biases, by for example incorporating blinded assessments in aware participants or by including more objective cognitive tasks or digital biomarkers such as passive monitoring of speech or eye-tracking.

Interestingly, in non-carriers, awareness was not associated with cognitive change but was linked to higher longitudinal depressive symptom trajectories, indicating that learning one’s non-carrier status may also have emotional consequences. At baseline, they experience fewer depressive symptoms compared to unaware non-carriers, but over time, their depressive symptoms start to increase. Possible explanations might be the sense of *survivor’s guilt*, which is also described in HD families [37], or feeling emotionally or socially distant from affected relatives. Furthermore, in a qualitative study of non-carriers of HD, some non-carriers reported that they felt psychological pressure to ‘do something extraordinary’ in their lives [38]. Together, these findings suggest that a negative genetic result does not guarantee psychological relief. Understanding these reactions is essential for providing appropriate counseling and ensuring that non-carriers receive adequate psychological support following disclosure as well.

Cross-sectionally, awareness of genetic status was not associated with cognitive test performance or behavioral symptoms in neither carriers nor non-carriers. One could have assumed that these effects were caused by the variation in timing of disclosure in our sample: some received genetic counseling years before their baseline assessment, while others learned their status shortly beforehand or during the study. However, our analyses showed no baseline differences between recently informed and longer-aware participants. Nevertheless, variability in disclosure timing may still have reduced sensitivity to subtle effects.

The subgroup of participants who learned their genetic status during the study provided a unique opportunity to examine within-person changes before and after disclosure. Examining the visit immediately before and after disclosure, non-carriers showed more depressive symptoms before knowing their genetic status than following disclosure. This may reflect feelings of relief, as also reported by participants in the study by Graafland et al. [5]. However, our findings did not reveal significant differences in outcomes before and after disclosure of genetic status over time. This contrasts with previous studies that have reported short-term psychological effects following disclosure of genetic status [10–13]. Notably, our study included a greater number of follow-up assessments over a longer period, which may partly account for these discrepant findings. Still, these results must be interpreted with caution, as the sample size was small, limiting statistical power, and disclosure occurred at different follow-up visits for participants, introducing variability in the pre- and post-learning intervals that may further reduce the sensitivity of the analysis. Consequently, it remains unclear whether disclosure meaningfully influences these trajectories, underscoring the need for larger sample sizes and more robust longitudinal designs to address this question.

A strength of this study is its large and longitudinal cohort of genetic FTD pathogenic variant carriers, matched with pathogenic-negative controls. In addition, awareness of status was always based on genetic testing. As (monogenetic) FTD is a rare neurodegenerative disease, one limitation still lies in the small sample sizes after splitting into subgroups, which affected the generalizability of our findings and reduced statistical power. This also limited the possibility to explore differences in trajectories between pathogenic variants. The distribution of genetic subgroups also differed between aware and unaware carriers, which may have complicated the interpretation of our findings and further limited their generalizability as specific pathogenic variants are associated with distinct phenotypic profiles and disease courses. Moreover, most behavioral and neuropsychiatric assessments used were caregiver-based and therefore prone to subjectivity. Furthermore, repeated cognitive testing in longitudinal studies could induce practice and familiarity effects that may mask subtle decline or improvement in cognitive outcomes. We partially accounted for this in our study by using parallel versions of tests when possible and controlling for parallel versions in our sensitivity analysis. However, while the use of parallel test versions helps mitigate familiarity effects, it may also introduce additional measurement noise due to subtle differences in difficulty or effects between versions. Notably, when we adjusted for parallel test versions in our analyses, several observed associations were attenuated, with some effects no longer reaching statistical significance. This could suggest that part of the initially observed effects may have been influenced by version-related variability, although reduced precision due to inclusion of an additional covariate may also have contributed. Lastly, clinical variables such as lived experiences with the disease, socioeconomic status, or coping style may moderate the impact of disclosure [39], but were not assessed in this study. Overall, while the strengths enhance the relevance of our findings, the limitations highlight the need for larger and methodologically comprehensive studies to fully understand the impact of genetic status awareness in FTD.

Taken together, among carriers, awareness of genetic status was not associated with worse cognitive, behavioral, or neuropsychiatric symptoms at a single time point, but did influence longitudinal changes in cognitive outcomes. This could reflect both true disease-related change and psychological effects of awareness, such as stress or expectancy, which can affect performance on demanding cognitive tests. Awareness-related bias in clinical ratings, including the CDR^®^-plus-NACC-FTLD [16], may lead to overestimation of impairment, potentially resulting in presymptomatic individuals being classified as impaired and being prematurely included in clinical trials. In non-carriers, increasing depressive symptoms may reflect survivor’s guilt or the need to reorient one’s life. These findings highlight the importance of considering genetic awareness in research, trial design, and genetic counseling. Study limitations point to the need for larger, more homogeneous samples, better control of rater and awareness-related bias, and designs that distinguish psychological effects of disclosure from true cognitive decline.

## Supplementary Information

Below is the link to the electronic supplementary material.

Supplementary file1 (DOCX 34 KB)

Supplementary file2 (DOCX 796 KB)

## Data Availability

Data not provided in the article may be shared (anonymized) at the request of a qualified investigator for purposes of replicating procedures and results.
