# Supplementary material for: Impact of awareness of genetic status on cognitive, behavioral, and neuropsychiatric outcomes in genetic frontotemporal dementia
Source: J Neurol. 2026 Jun 12;273(7):393. doi: 10.1007/s00415-026-13892-0 (PMC13263297; doi:10.1007/s00415-026-13892-0)

**Suppl. Figure 1.** Baseline scores on NPI-Q subdomains in all groups


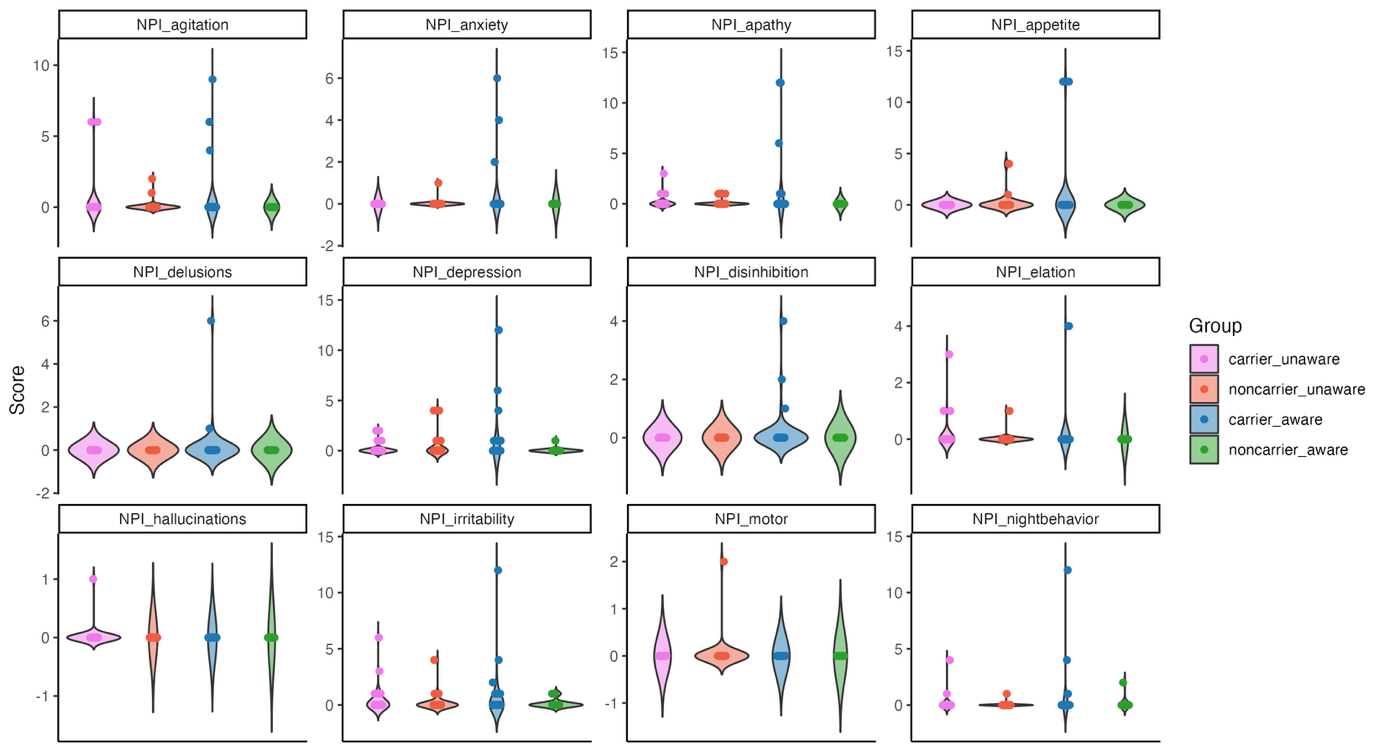


**Suppl. Figure 2.** Longitudinal trajectories in NPI-Q subdomains in carriers


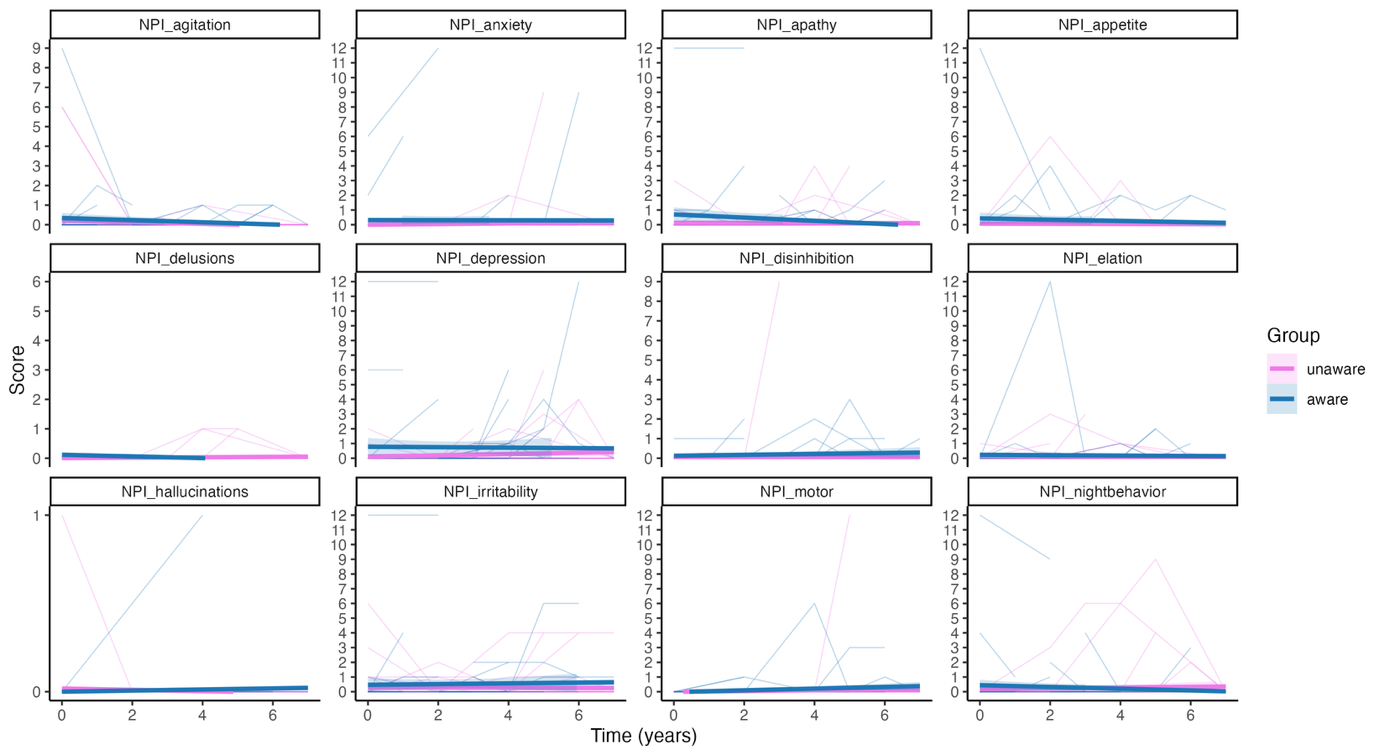


**Suppl. Figure 3.** Longitudinal trajectories in NPI-Q subdomains in non-carriers


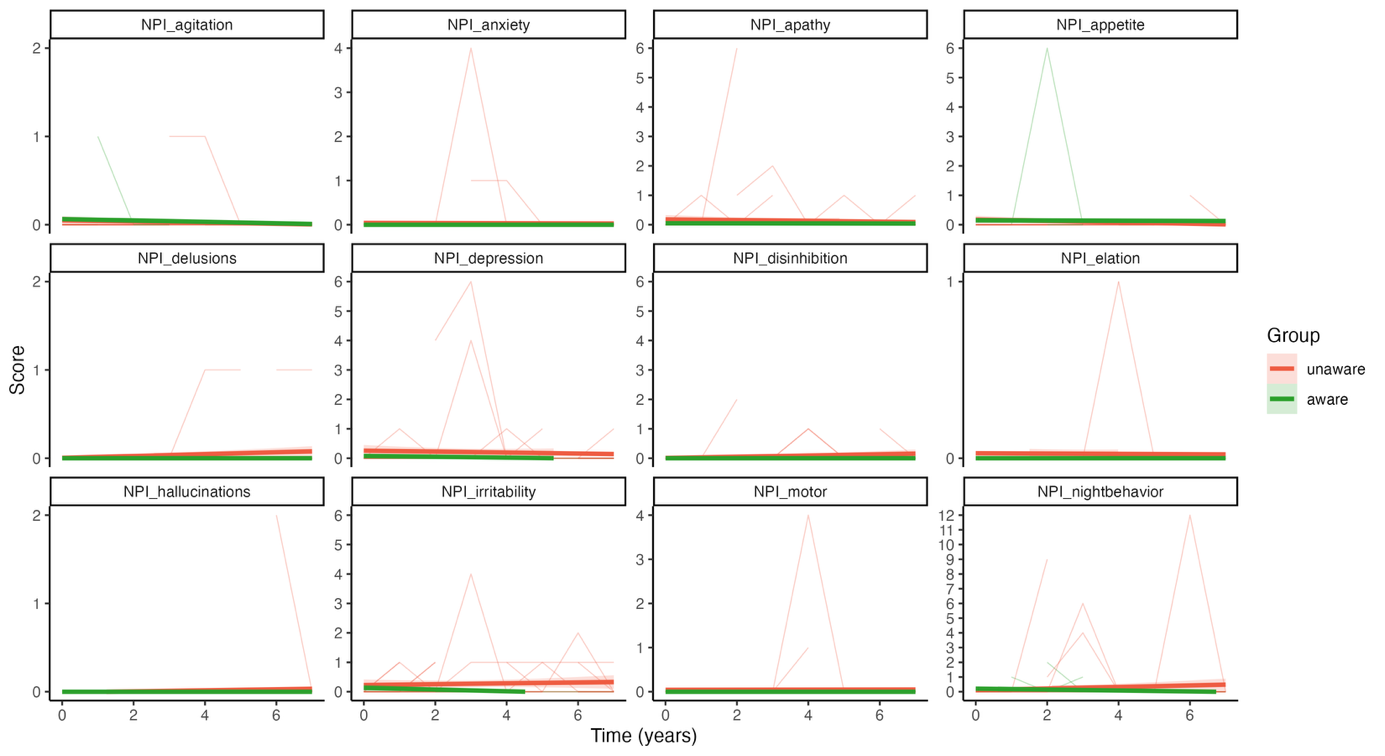


**Suppl. Figure 4.** Trajectories (95% CI) pre- and post-awareness NPI-Q subdomains with a maximum of 3 follow-ups pre- and -post learning genetic status


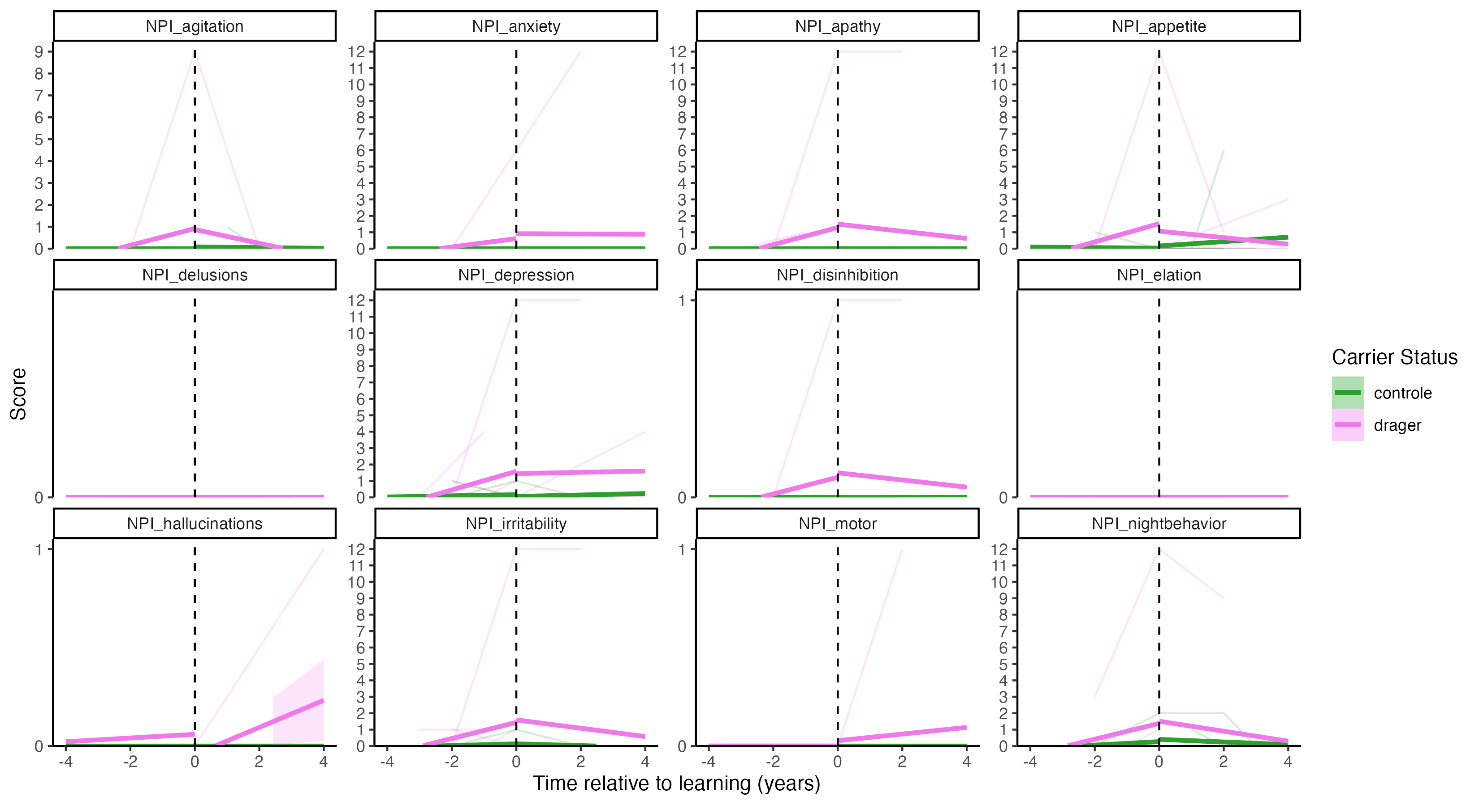

Supplement: Supplementary file 2 — Supplementary file2 (DOCX 796 KB) [file 415_2026_13892_MOESM2_ESM.docx]
